# Genome-Wide Linkage Mapping of QTL for Adult-Plant Resistance to Stripe Rust in a Chinese Wheat Population Linmai 2 × Zhong 892

**DOI:** 10.1371/journal.pone.0145462

**Published:** 2015-12-29

**Authors:** Jindong Liu, Zhonghu He, Ling Wu, Bin Bai, Weie Wen, Chaojie Xie, Xianchun Xia

**Affiliations:** 1 Institute of Crop Science/National Wheat Improvement Center, Chinese Academy of Agricultural Sciences, Beijing, China; 2 Department of Plant Genetics & Breeding/State Key Laboratory for Agrobiotechnology, China Agricultural University, Beijing, China; 3 International Maize and Wheat Improvement Center (CIMMYT) China Office, Chinese Academy of Agricultural Sciences, Beijing, China; 4 Crop Research Institute, Sichuan Academy of Agricultural Sciences, Chengdu, Sichuan, China; 5 Wheat Research Institute, Gansu Academy of Agricultural Sciences, Lanzhou, Gansu, China; 6 College of Agronomy, Xinjiang Agricultural University, Urumqi, Xinjiang, China; USDA, UNITED STATES

## Abstract

Stripe rust is one of the most devastating diseases of wheat (*Triticum aestivum*) worldwide. Adult-plant resistance (APR) is an efficient approach to provide long-term protection of wheat from the disease. The Chinese winter wheat cultivar Zhong 892 has a moderate level of APR to stripe rust in the field. To determine the inheritance of the APR resistance in this cultivar, 273 F_6_ recombinant inbred lines (RILs) were developed from a cross between Linmai 2 and Zhong 892. The RILs were evaluated for maximum disease severity (MDS) in two sites during the 2011–2012, 2012–2013 and 2013–2014 cropping seasons, providing data for five environments. Illumina 90k SNP (single nucleotide polymorphism) chips were used to genotype the RILs and their parents. Composite interval mapping (CIM) detected eight QTL, namely *QYr*.*caas-2AL*, *QYr*.*caas-2BL*.*3*, *QYr*.*caas-3AS*, *QYr*.*caas-3BS*, *QYr*.*caas-5DL*, *QYr*.*caas-6AL*, *QYr*.*caas-7AL* and *QYr*.*caas-7DS*.*1*, respectively. All except *QYr*.*caas-2BL*.*3* resistance alleles were contributed by Zhong 892. *QYr*.*caas-3AS* and *QYr*.*caas-3BS* conferred stable resistance to stripe rust in all environments, explaining 6.2–17.4% and 5.0–11.5% of the phenotypic variances, respectively. The genome scan of SNP sequences tightly linked to QTL for APR against annotated proteins in wheat and related cereals genomes identified two candidate genes (autophagy-related gene and disease resistance gene *RGA1*), significantly associated with stripe rust resistance. These QTL and their closely linked SNP markers, in combination with kompetitive allele specific PCR (KASP) technology, are potentially useful for improving stripe rust resistances in wheat breeding.

## Introduction

Stripe rust (yellow rust, YR), caused by *Puccinia striiformis* f. sp. *tritici* (*Pst*), is a very destructive fungal disease of common wheat (*Triticum aestivum*), and it is prevalent in temperate or medium altitude and maritime wheat-growing regions, such as China, India, Pakistan, Australia, USA, Mexico, and northwestern Europe [1,2]. Yield losses caused by YR ranged from 10 to 70% and over 20 significant YR epidemics were documented worldwide during 1954–2010 [3]. During recent years, YR has occurred in about 4.2 million ha and caused heavy yield losses in the southwestern and northwestern China annually [4–6].

Although YR can be controlled by fungicides, this may be limited by management and financial constraints. Resistant cultivars are an economically effective and environmentally friendly approach to control the disease [7]. Resistance to YR can be categorized broadly into all-stage resistance and adult-plant resistance. Generally, all-stage resistance is conferred by major genes that are race-specific in effect and qualitatively inherited [8–10]. However, such resistance is usually not durable and readily overcome by new pathogen races. A singly deployed all-stage resistance gene is effective only for about 3–5 years on average [8]. In contrast, adult-plant resistance (APR) is more likely conferred by minor genes that are typically race non-specific, inherited quantitatively, and has greater potential for durability [11–12]. An APR gene usually contributes partial resistance and combinations of 4–5 APR genes act additively to confer adequate levels of durable resistance [13–14]. For example, *Yr18* and 2–4 additional minor genes have provided effective resistance to YR in China and other countries for over 80 years [15–17]. APR is being increasingly emphasized in breeding for rust resistance worldwide [18–19] mainly because of its potential but not exclusive durability [20].

To date, 70 YR resistance genes at 67 wheat loci have been formally catalogued [18–21]. Most of these genes are race-specific, and in China, the majorities have been overcome by new races [22]. APR genes at 13 loci have been cataloged, namely, *Yr16* [23], *Lr34/Yr18/Pm38/Sr57* [24–25], *Lr46/Yr29/Pm39/Sr58* [26–27], *Sr2/Yr30* [28], *Yr36* [29–30], *Yr39* [31], *Lr67/Yr46/Pm46/Sr55* [32], *Yr48* [33], *Yr49* [34], *Yr52* [35], *Yr54* [36], *Yr59* [37], and *Yr62* [38]. Some such as *Lr34/Yr18/Pm38/Sr57*, *Lr46/Yr29/Pm39/Sr58*, *Sr2/Yr30* and *Lr67/Yr46/Pm46/Sr55* confer pleiotropic disease resistances. *Lr34/Yr18/Pm38/Sr57* [30], *Yr36* [15], and *Lr67/Yr46/Pm46/Sr55* (Lagudah, pers. comm.) were cloned and appear to have quite different molecular structures to the currently cloned all-stage rust resistance genes.

During the last 15 years more than 160 QTL that reduce YR severity were assigned to 49 chromosomal regions [18,21]. Even allowing for commonality this represents a high level of genetic diversity. Given that combinations of several such QTL (genes) are required to obtain sufficiently high levels of resistance [7,14], the expected reward is durability. Many studies have shown that such resistance can be obtained by visual selection in disease nurseries, but clearly such selection is greatly aided (perhaps even circumvented) by use of molecular markers. Molecular markers can be used in programs that aim to combine both APR and all-stage resistance where it is impossible or extremely difficult to visually assess APR effects in the presence of all-sage resistance genes.

Marker platforms used in the past for linkage map construction and QTL mapping included restriction fragment length polymorphism (RFLP) [7,39–40], amplified fragment length polymorphism (AFLP) [41–42], simple sequence repeats (SSRs) [43–45] and diversity arrays technology (DArT) [22,46]. However, the low level of polymorphism and the large genome size of common wheat ultimately limits mapping resolution. SNP arrays that provide a large number of genome-wide polymorphic, co-dominant markers for high-throughput, cost-effective genotyping are ideal for QTL mapping. The high-density linkage maps constructed with SNP markers can be used for high-resolution QTL analysis and identification of candidate genes associated with quantitative traits [47–48]. The recently developed wheat 90K SNP array, comprising 81,587 SNPs with a dense coverage of the wheat genome [49], can be used for efficient QTL mapping and construction of high-density maps [47–48,50].

Zhong 892 is a good semi-dwarf winter wheat line, exhibiting a moderate level of resistance to YR and powdery mildew in the field, whereas it is susceptible at the seedling stage, indicating a typical APR. However, little is known about the inheritance of resistance to YR in this cultivar. The objectives of the current study were to identify APR QTL to YR in a Linmai 2 × Zhong 892 RIL population using high-density SNP markers, and to assess the stability of detected QTL across environments.

## Materials and Methods

### Plant materials

A total of 273 F_2:6_ RILs were developed from the Linmai 2 × Zhong 892 cross. Zhong 892 and Linmai 2 were highly susceptible to currently prevalent *Pst* races CYR29, CYR31, CYR32, and CYR33 at the seedling stage, whereas they showed moderately resistant and moderately susceptible, respectively, at the adult-plant stage in the field. The RILs were generated through single seed descent, where one random spike was harvested in each generation and advanced to the next generation.

### Field trials

The F_2:6_ RILs and their parents were evaluated for APR to YR at the Pixian experimental station of Sichuan Academy of Agricultural Sciences (30°05′N, 102°54′E) in Sichuan province by Dr. Ling Wu (a co-author of this manuscript, and a wheat breeder in Sichuan Academy of Agricultural Sciences), and the Qingshui experimental station of Gansu Academy of Agricultural Sciences (34°05′N′, 104°35′E) in Gansu province by Dr. Bin Bai (a co-author of this manuscript, and a wheat breeder in the Qingshui experimental station) during the 2011–2012, 2012–2013 and 2013–2014 cropping seasons, providing data for five environments. Both locations are hotspots for YR in China with ideal conditions for rust infection and spread. Field trials were conducted in randomized complete blocks with three replicates at each location. Each plot consisted of a single row with 1.5 m length and 25 cm between rows. Approximately 50 seeds were sown in each row. Every tenth row was planted with the highly susceptible control cv. Huixianhong. To ensure ample field inoculum, infection rows of cv. Chuanyu 12 and Huixianhong surrounded the experimental areas at Pixian and Qingshui, respectively. Inoculations at both sites each year were performed at the three-leaf stage with a mixture of prevalent Chinese *Pst* races, CYR29, CYR31, CYR32 and CYR33, using spray method (around Jan. 5 at Pixian and April 10 at Qingshui). Maximum disease severities (MDS) [51] of RILs were scored 18–20 d post-flowering, when YR severities on the control Huixianhong reached a maximum level around 8–10 April at Pixian and 7–10 June at Qingshui.

### Genotyping

Genomic DNA was extracted from five bulked leaves each line using a modified CTAB procedure [52]. All 273 lines and their parents were genotyped with the Illumina 90K iSelect assay [49] by Capital Bio Corporation (Beijing, China; http://www.capitalbio.com). Genotypic clusters for each SNP were determined using the manual option of Genome Studio version 1.9.4 with the polyploid clustering version 1.0.0 (Illumina; http://www.illumina.com), based on data from all genotypes. The default clustering algorithm implemented in Genome Studio was initially used to classify each SNP call into three distinct clusters corresponding to the AA, BB and AB genotypes expected for bi-allelic SNPs. These SNP markers were described by Wang et al. [49] and only co-dominant SNP markers were used for genetic mapping. The chromosome location of each SNP was based on wheat SNP consensus map [49].

### Statistical analysis

The MDS were evaluated in five environments during three cropping seasons, and data from each environment and the arithmetic means for each line were used for analysis of variance (ANOVA) and subsequent QTL mapping. ANOVA and computation of correlation coefficients were performed by the SAS V9.0 (SAS Institute Inc., Cary, NC). The contributions of lines (RILs) and environments were evaluated by PROC MIXED, where environments were treated as fixed effects, and lines, line × environment interaction and replicates nested in environments were all treated as random. The information in the ANOVA table was used to calculate broad sense heritability (*h*_*b*_^*2*^) for YR: *h*_*b*_^*2*^ = *σ*_*g*_^*2*^ */*(*σ*_*g*_^*2*^ *+ σ*_*ge*_^*2*^ */r + σ*_*ε*_^*2*^ */re*), where *σ*_*g*_^*2*^, *σ*_*ge*_^*2*^ and *σ*_*ε*_^*2*^ were estimates of genotypic, genotype (line) × environment interaction and residual error variances, respectively, and *e* and *r* were the numbers of environments and replicates per environment.

### Genetic linkage map construction and QTL analysis

The genotypic data for SNP markers were used to construct genetic linkage maps with the software Joinmap V4.0 (http://www.kyazma.com) [53] and maps were made by MapChart V2.2 (http://www.earthatlas.mapchart.com) [54]. Map distances (in centimorgans, cM) were calculated based on the Kosambi mapping function [55].

Composite interval mapping (CIM) was performed using the software QTL Cartographer V2.5 (http://statgen.ncsu.edu/qtlcart/WQTLCart.htm) [56]. The walking speed chosen for all QTL was 2.0 cM, with *P* = 0.001 in stepwise regression. Based on 2,000 permutations at a probability of 0.01, the LOD score to declare significant QTL for MDS was 2.0–2.5 in all five environments and the averaged data, thus the LOD score 2.5 was set as the threshold for declaring significant QTL. The proportion of phenotypic variance (*R* ^*2*^) explained by a single QTL was determined by the square of the partial correlation coefficient, and the total *R* ^*2*^ in a simultaneous fit was calculated through multiple linear regressions using the SAS REG procedure (SAS Institute Inc., Cary, NC). Individual environment QTL overlapping within a 20 cM interval were considered common. In this study, the genotype of Zhong 892 was defined as 2, and the genotype of Linmai 2 was defined as 0. Thus, the allele from Zhong 892 reduced YR MDS when the additive effect was negative. QTL detected in at least two environments were included in the results.

### Search for candidate genes for stripe rust resistance

In order to identify candidate genes involved in QTL for stripe rust resistance detected in the Linmai 2/Zhong 892 population, the EST sequences (about 50 bp upstream and 50 bp downstream) corresponding to the SNP markers [49] located in the regions underlying the QTL were used to BLAST against the NCBI nucleotide database (http://www.ncbi.nlm.nih.gov/) and European Nucleotide Archive (http://www.ebi.ac.uk/ena). BLAST hits were filtered to an *e*-value threshold of 10^−5^ with an identity higher than 75%.

## Results

### Phenotypic evaluation

The mean MDS of the susceptible control Huixianhong were over 80% across all environments. The averaged MDS for the 273 RILs were 48.3%, 44.5%, 56.6%, 35.2% and 42.8%, ranging between 5.0–95.1%, 2.0–90.0%, 3.1–96.2%, 3.1–74.5% and 2.6–84.2% in Pixian 2012, Pixian 2013, Pixian 2014, Qingshui 2013 and Qingshui 2014, respectively (S1 Fig), indicating polygenic variation. Zhong 892 was rated with a mean MDS of 23.3%, 34.6%, 40.7%, 25.0% and 20.0% in Pixian 2012, Pixian 2013, Pixian 2014, Qingshui 2013 and Qingshui 2014, respectively, whereas Linmai 2 had mean MDS of 53.2%, 46.8%, 60.2%, 38.3% and 42.7% in the five environments, respectively (S1 Fig). The MDS were significantly correlated (0.47–0.61, *P* < 0.01) across environments, and the broad-sense heritability of YR MDS was 0.85. ANOVA of MDS revealed significant differences (*P* < 0.01) among RILs, environments, and line × environment interactions (Table 1).

**Table 1:** Analysis of variance of MDS for stripe rust response in the Linmai 2 × Zhong 892 RIL population.

Source of variance	*Df*	Mean square	*F* value
**Replicate (environment)**	2	1571	8.8**
**Environment**	4	38028	212.0**
**Line**	272	3125	17.4**
**Line × Environment**	1088	311	1.7**
**Error**	1654	179	

** Significant at *P* < 0.0001

### QTL for APR to YR

**Fig 1:**
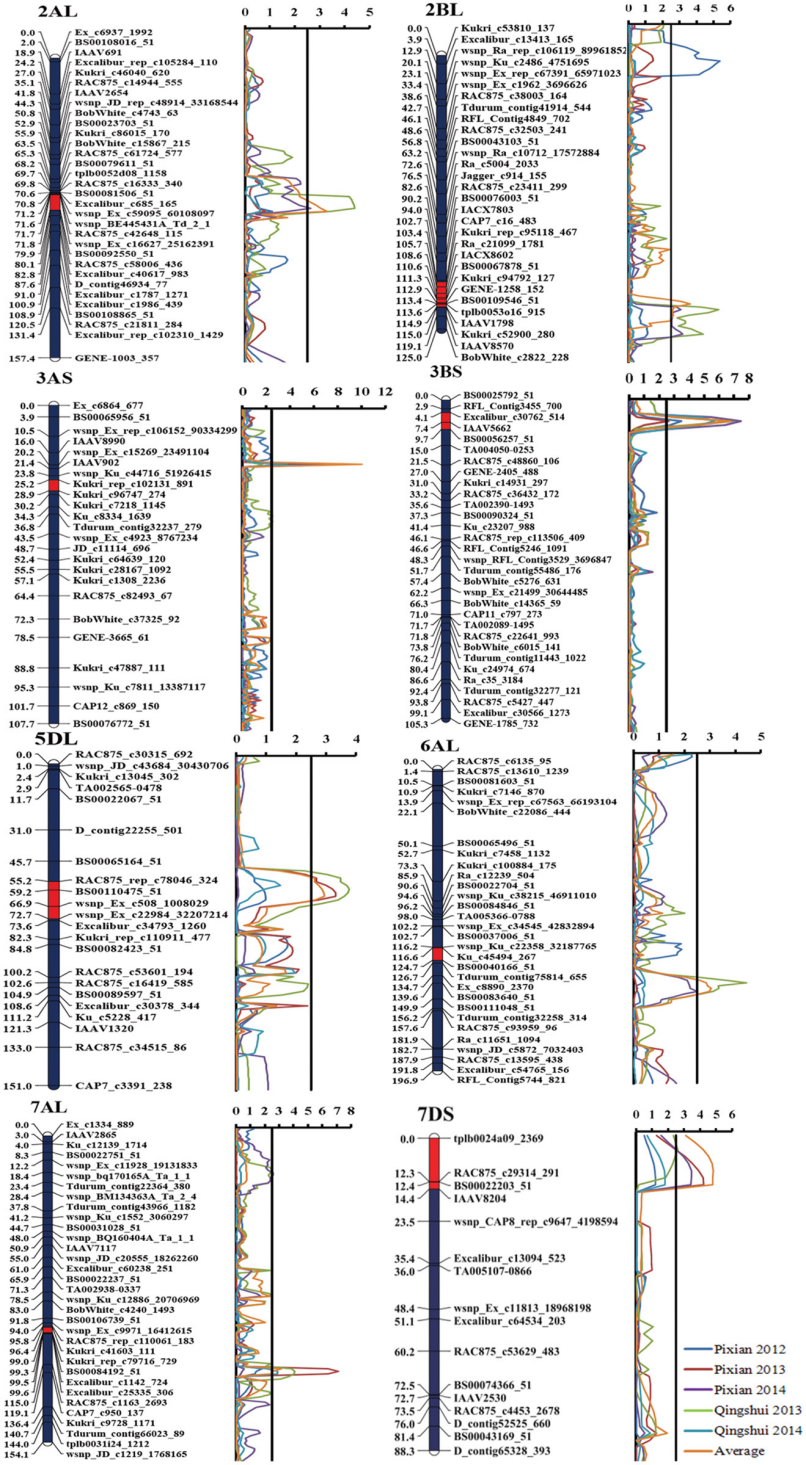
LOD contours obtained by composite interval mapping of QTL for stripe rust response in the Linmai 2 × Zhong 892 RIL population. Pixian 2012, Pixian 2013, Pixian 2014, Qingshui 2013, Qingshui 2014, and averaged MDS are indicated with deep blue, red, purple, green, light blue and orange colours. LOD thresholds of 2.5 are indicated by solid vertical lines.

Eight QTL were identified on different chromosomes, namely *QYr*.*caas-2AL*, *QYr*.*caas-2BL*.*3*, *QYr*.*caas-3AS*, *QYr*.*caas-3BS*, *QYr*.*caas-5DL*, *QYr*.*caas-6AL*, *QYr*.*caas-7AL* and *QYr*.*caas-7D*S.1 (Table 2; Fig 1). The resistance alleles of the QTL on 2AL, 3AS, 3BS, 5DL, 6AL, 7AL and 7DS were contributed by Zhong 892, whereas *QYr*.*caas-2BL*.*3* was from Linmai 2.

**Table 2:** QTL for stripe rust response in the Linmai 2 × Zhong 892 RIL population across five environments.

QTL^a^	Closest marker	Distance (cM)	Pixian 2012			Pixian 2013			Pixian 2014			Qingshui 2013			Qingshui 2014			Average		
			LOD^b^	*R* ^*2*^ ^c^	Add^d^	LOD	*R* ^*2*^	Add	LOD	*R* ^*2*^	Add	LOD	*R* ^*2*^	Add	LOD	*R* ^*2*^	Add	LOD	*R* ^*2*^	Add
***QYr*.*caas-2AL***	*BS00092550_51*	1.6							2.6	4.4	-3.7	4.4	7.2	-7.9				3.0	5.1	-5.0
***QYr*.*caas-2BL*.*3***	*IACX8602*	0.9							3.0	5.6	3.9	5.3	7.6	9.7				3.4	5.9	4.9
***QYr*.*caas-3AS***	*Kukri_c96747_274*	1.5	5.4	9.2	-8.1	3.7	6.2	-5.9	8.1	13.0	-5.2	10.8	17.4	-11.8	3.7	6.5	-3.1	10.0	15.8	-6.0
***QYr*.*caas-3BS***	*IAAV5662*	1.1	3.5	5.9	-6.9	2.6	5.0	-3.1	7.4	11.5	-5.1	6.9	10.5	-10.6	5.7	9.4	-3.8	6.8	10.5	-5.5
***QYr*.*caas-5DL***	*wsnp_Ex_c508_1008029*	1.7				3.4	5.8	-6.4				3.8	6.5	-10.7				2.9	4.7	-4.5
***QYr*.*caas-6AL***	*Ku_c45494_267*	0.8							2.6	4.3	-4.0	4.6	7.8	-9.7				3.0	4.9	-4.2
***QYr*.*caas-7AL***	*Excalibur_c25335_306*	1.2				7.2	12.0	-8.3				4.1	7.7	-8.0	2.9	5.0	-3.0	3.1	6.6	-4.6
***QYr*.*caas-7DS*.*1***	*RAC875_c29314_291*	2.3				4.2	7.8	-5.8	3.5	6.7	-4.9	2.7	5.7	-3.7				4.8	9.1	-4.6
***Total R*** ^***2***^ ^e^				10.2			33.4			36.9			42.8			18.3			48.7	

^a^ QTL were detected with a LOD threshold 2.5 for declaring QTL based on 2,000 permutations at *P* = 0.01.

^b^ Logarithm of odds score.

^c^ Percentage of phenotypic variance explained by the QTL.

^d^ Additive effect of resistance allele.

^e^ The total *R* ^*2*^ in a simultaneous fit was calculated through multiple linear regressions.

A major and consistent QTL for YR resistance, *QYr*.*caas-3AS*, was flanked by *Kukri_rep_c102131_891* and *Kukri_c96747_274* with genetic distances of 2.2 and 1.5 cM, respectively, and explained 9.2%, 6.2%, 13.0%, 17.4%, 6.5% and 15.8% of the phenotypic variances in Pixian 2012, Pixian 2013, Pixian 2014, Qingshui 2013 Qingshui 2014 and the averaged MDS, respectively. The second consistently detected QTL with a relatively large effect, *QYr*.*caas-3BS*, between *IAAV5662* and *BS00056257_51* with genetic distances of 1.1 and 1.2 cM, respectively, explained 5.0 to 11.5% of the phenotypic variances in five environments and the averaged MDS. The third QTL, *QYr*.*caas-7AL*, between *Kukri_c41603_111* and *Excalibur_c25335_306* with genetic distances of 2.0 and 1.2 cM, respectively, accounted for 12.0%, 7.7%, 5.0% and 6.6% of the phenotypic variances in Pixian 2013, Qingshui 2013, Qingshui 2014, and the averaged MDS, respectively. The fourth QTL, *QYr*.*caas-7DS*.*1*, between *tplb0024a09_2369* and *RAC875_c29314_291* with genetic distances of 10.0 and 2.3 cM, respectively, explained 7.8%, 6.7%, 5.7% and 9.1% of the phenotypic variances in Pixian 2013, Pixian 2014, Qingshui 2013, and the averaged MDS, respectively (Table 2; Fig 1).

Three QTL, *QYr*.*caas-2AL*, *QYr*.*caas-2BL*.*3* and *QYr*.*caas-6AL*, were found in Pixian 2014, Qingshui 2013 and the averaged MDS, and explained 4.4–7.2%, 5.6–7.6% and 4.3–7.8% of the phenotypic variances, respectively (Table 2; Fig 1). *QYr*.*caas-2AL* was flanked by *wsnp_Ex_c16627_25162391* and *BS00092550_51* with genetic distances of 6.5 and 1.6 cM, respectively; *QYr*.*caas-2BL*.*3* was located in the interval of *Ra_c21099_1781* and *IACX8602* with genetic distances of 2.0 and 0.9 cM, respectively; *QYr*.*caas-6AL* was mapped on chromosome 6AL between *Ku_c45494_267* and *BS00040166_51* with genetic distances of 0.8 and 7.3 cM, respectively (Fig 1).

*QYr*.*caas-5DL* flanked by *wsnp_Ex_c508_1008029* and *wsnp_Ex_c22984_32207214* with genetic distances of 1.7 and 4.1 cM, respectively, explained 5.8%, 6.5% and 4.7% of the phenotypic variances in Pixian 2013, Qingshui 2013, and the averaged MDS, respectively (Table 2; Fig 1).

**Fig 2:**
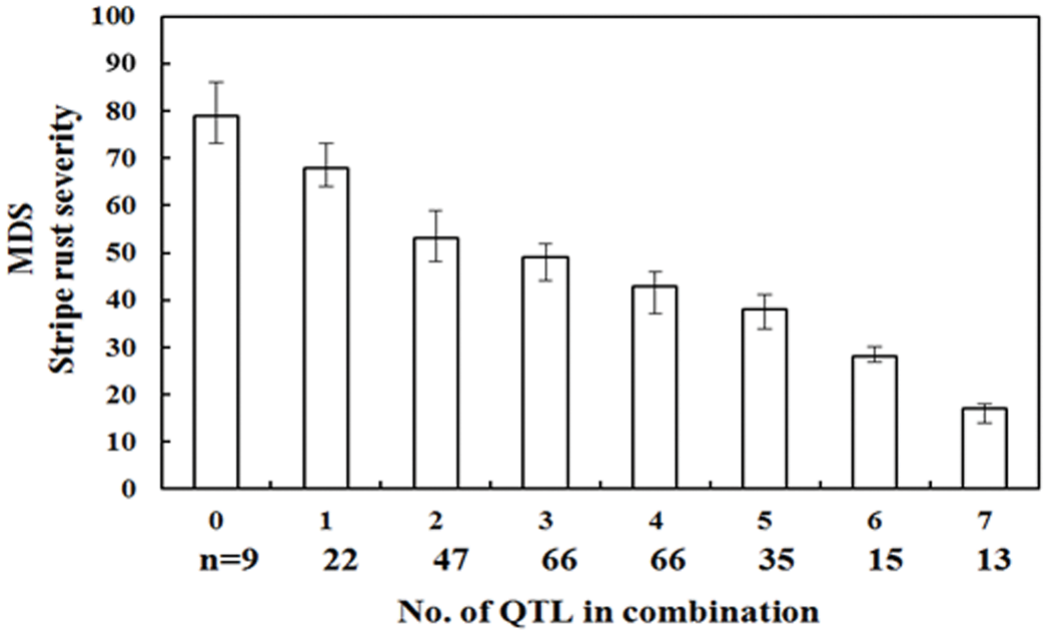
The effects of QTL combinations on stripe rust severity among different classes. The number in abscissa indicates the number of QTL combined in each subset of RILs. The error bars indicate the standard error of sample means.

To identify the combined effects of these QTL, the flanking markers were used to select RILs possessing the corresponding QTL. Among 42 combinations (genotypes) of eight resistance QTL, a significant additive effect for stripe rust was found in the RILs possessing 5–7 QTL (S1 Table). Results indicated that the more resistance genes a line possessed, the lower the disease severity was (Fig 2). When 5–7 genes were combined in a line, the MDS was less than 30% on average (Fig 2).

## Discussion

### Comparisons of QTL with previous reports

#### QYr.caas-3AS

The QYr.caas-3AS was derived from Zhong 892, and it was detected consistently across all environments. A number of QTL were previously found on chromosome 3A [57–59]. As this is a first report of a QTL for YR resistance on chromosome 3AS, it is likely to be a new gene.

#### QYr.caas-3BS

Several QTL for YR resistance on chromosome 3BS were reported previously [24,27,33,40,42,60–61]. Most of these QTL are probably in the same region [57]. For example, loci *Xfba190* [40], and *Xgwm493* [60] are close to *Xgwm389* and *Xgwm533* [24]. *Yr30* [40], *Sr2* [62], pseudo-black chaff [63] and a Fusarium head blight resistance gene *Fhb1* [64] were all closely linked to *Xgwm533*. *QYr*.*caas-3BS* derived from Zhong 892 showed consistent resistance to YR across all environments. The SNP markers closely linked to *QYr*.*caas-3BS* were *IAAV5662* and *BS00056257_51*. SSR marker *Xgwm533* and SNP marker *BS00056257_51* belong to the same bin (3BS9-0.57–0.78). The molecular marker for *csSr2* [65] was not present in Zhong 892 indicating absence of *Sr2/Yr30*. Nevertheless, *QYr*.*caas-3BS* was likely to be the same as some of the QTL described above. Further study is needed to test the allelism between QYr.caas-3BS and other previously reported QTL.

#### QYr.caas-5DL

Suenaga et al. [24] identified *QYr*.*jirc-5DL* on chromosome 5DL closely linked to SSR locus *Xwmc215*; this explained 3.9% of the phenotypic variance. Imtiaz et al. [66] found *QYr*.*nsw-5DL* on chromosome 5DL, closely linked to *Xgwm583*, explaining 6.1% of the phenotypic variance. The SSR markers *Xwmc215* and *Xgwm583*, and the SNP marker *wsnp_Ex_c22984_32207214* belong to the same bin (5DL5-0.76–1.00). Thus, *QYr*.*caas-5DL* was likely to be the same as the QTL described previously.

#### QYr.caas.7AL

Dedryver et al. [42] identified an APR QTL *QYr*.*inra-7A* on chromosome 7A in wheat cultivar Recital; it was located between AFLP markers *Xbcd129b* and *Xfba127c*. Zwart et al. [67] identified *QYr*.*sun-7A* on chromosome 7A in wheat cultivar CPI133872; this was mapped between AFLP markers *Wpt-7214* and *Wpt-4877*. Due to different kinds of markers used in those and current studies, it is difficult to determine whether they are the same or not.

#### QYr.caas-7DS.1

The pleiotropic APR gene *Lr34/Yr18/Pm38* located on the short arm of chromosome 7D [68–70], and closely linked to *Xgwm295*. Many other studies also detected QTL in this region, explaining 20–40% of the phenotypic variances [16,24,40,67,71–76]. The closest marker *BS00022203_51* linked to *QYr*.*caas-7DS*.*1* in bin 7DS5-0.36–0.61 is near *Xgwm295* in bin 7DS4-0.61–1.00. The available information indicates that *QYr*.*caas-7DS*.*1* is different from *Yr18*. Firstly, tests with the STS marker *csLv34* [25] were negative and secondly, *Lr34/Yr18/Pm38* is linked with the phenotypic marker LTN (leaf tip necrosis), which was not observed in the present materials under conditions where LTN was clearly expressed in other materials. In addition, the effect of *QYr*.*caas-7DS*.*1* on YR response was much less than normally observed on materials with *Yr18*.

### Candidate genes related to stripe rust resistance

With the rapid development of gene chip technologies in wheat, the SNP markers play more and more important role in the development of high-density genetic linkage maps [77] and genetic diversity studies [78]. The wheat 90K SNP arrays were mainly developed from expressed genes, and the availability of EST sequence data corresponding to SNP markers makes it possible to identify candidate genes by BLAST against the database of common wheat, *Brachypodium* and other cereals genome sequences.

The bioinformatics analysis of SNP markers tightly linked to stripe rust resistance QTL indicated that the closest marker IACX8602 for *QYr*.*caas-2BL*.*3* corresponded to the autophagy-related gene [79]. The autophagy-related proteins ATG4 and ATG8 are crucial for autophagy biogenesis and play important role in resistance response to fungus infection, such as *Blumeria graminis* f. sp. *tritici* [79]. Another SNP marker on chromosome 7DS (*RAC875_c29314_291*) corresponded to a putative disease resistance gene *RGA1* [80], at a distance of 2.3 cM from the LOD contour peak of *QYr*.*caas-7DS*. However, since the resistance response to fungus is a very complicated biological process, a more detailed experimental analysis should be carried out to confirm the role of these genes on stripe rust resistance.

### Potential application of QTL for MAS in wheat breeding

The present study indicated that any combination of 4–5 APR genes with minor or intermediate effects in a line may provide a higher level of resistance to YR, which is consistent with previous reports [7,22,45,81]. In all QTL combinations, *QYr*.*caas-3AS* and *QYr*.*caas-3BS* showed high and stable resistance than the others, and their additive effects played more important role than interaction effects in this study (S1 Table).

QTL identified across multiple environments should be useful for marker-assisted selection (MAS) [82]. In the present study, *QYr*.*caas-3AS*, *QYr*.*caas-3BS*, *QYr*.*caas-7AL* and *QYr*.*caas-7DS* showed consistent effects across multiple environments. *QYr*.*caas-3AS* and *QYr*.*caas-3BS* were tightly linked to *Kukri_c96747_274* and *IAAV5662*, with genetic distances of 1.5 and 1.1 cM, respectively; they should provide accurate selection in wheat breeding. KASP is a uniplex SNP genotyping platform that offers cost-effective and scalable flexibility in applications that require small to moderate numbers of markers, such as marker-assisted selection, and QTL fine mapping [83]. The QTL reported in the present study, *QYr*.*caas-3AS*, *QYr*.*caas-3BS*, *QYr*.*caas-7AL* and *QYr*.*caas-7DS*, and their closely linked SNP markers *Kukri_c96747_274*, *IAAV5662*, *Excalibur_c25335_306* and *RAC875_c29314_291*, could be potentially used for MAS and pyramiding of stripe rust APR genes in wheat breeding using KASP technology.

## Supporting Information

S1 Fig Frequency distributions of MDS for stripe rust responses in the Linmai 2 × Zhong 892 RIL population. A, Pixian 2012; B, Pixian 2013; C, Pixian 2014; D, Qingshui 2013; E, Qingshui 2014; F, averaged MDS. Mean MDS for the parents, Linmai 2 and Zhong 892, are indicated by arrows.
(TIF)

S1 Table Mean stripe rust severity of lines with different QTL combinations from the Linmai 2/Zhong 892 RIL population. (DOCX)

## References

[1] Stubbs RW (1985) Stripe rust In: Roelfs AP, Bushnell WR. (eds) The cereal rusts II. Academic Press, Orlando, FL, pp61–101.

[2] Wan AM, Chen XM, He ZH (2007) Wheat stripe rust in China. Aust J Agric Res 58:605–619.

[3] Wellings CR (2011) Global status of stripe rust: a review of historical and current threats. Euphytica 179:129–141.

[4] He ZH, Lan CX, Chen XM, Zou YC, Zhuang QS, Xia XC (2011) Progress and perspective in research of adult-plant resistance to stripe rust and powdery mildew in wheat. Sci Agric Sin 44:2193–2215.

[5] National Agro-technical Extension and Service Center (NAESC) (2013) Epidemic characteristics of wheat stripe rust in China in 2013 and its control strategies. China Plant Prot 33:35–39.

[6] National Agro-technical Extension and Service Center (NAESC) (2014) Epidemic characteristics of wheat stripe rust in China in 2014 and its control strategies. China Plant Prot 33:47–52.

[7] Singh RP, Huerta-Espino J, Rajaram S (2000) Achieving near-immunity to leaf and stripe rusts in wheat by combining slow rusting resistance genes. Acta Phytopathol Entomol Hung 35:133–139.

[8] Johnson R (1981) Durable resistance: definition, genetic control, and attainment in plant breeding. Phytopathology 71:567–568.

[9] Chen XM, Line RF (1995) Gene action in wheat cultivars for durable, high-temperature, adult-plant resistance and interaction with race-specific, seedling resistance to Puccinia striiformis. Phytopathology 85:567–572.

[10] Carter AH, Chen XM, Garland-Campbell K, Kidwell KK (2009) Identifying QTL for high-temperature adult-plant resistance to stripe rust (Puccinia striiformis f. sp. tritici) in the spring wheat (Triticum aestivum L.) cultivar Louise. Theor Appl Genet 119:1119–1128. 10.1007/s00122-009-1114-2 19644666

[11] Johnson R (1984) A critical analysis of durable resistance. Annu Rev Phytopathol 22:309–330.

[12] Chen XM (2005) Epidemiology and control of stripe rust (Puccinia striiformis f. sp. tritici) on wheat. Can J Plant Pathol 27:314–337.

[13] Singh RP, Huerta-Espino J, William HM (2005) Genetics and breeding for durable resistance to leaf and stripe rusts in wheat. Turk J Agric For 29:121–127.

[14] Singh RP, Huerta-Espino J, Bhavani S, Herrera-Foessel SA, Singh D, Singh PK, et al (2011) Race-non-specific resistance to rust diseases in CIMMYT spring wheats. Euphytica 179:175–186.

[15] Krattinger SG, Lagudah ES, Spielmeyer W, Singh RP, Huerta-Espino J, McFadden H, et al (2009) A putative ABC transporter confers durable resistance to multiple fungal pathogens in wheat. Science 323:1360–1363. 10.1126/science.1166453 19229000

[16] Lu YM, Lan CX, Liang SS, Zhou XC, Liu D, Zhou G, et al (2009) QTL mapping for adult-plant resistance to stripe rust in Italian common wheat cultivars Libellula and Strampelli. Theor Appl Genet 119:1349–1359. 10.1007/s00122-009-1139-6 19756474

[17] Singh RP, Herrera-Foessel S, Huerta-Espino J, Singh S, Bhavani S, Lan CX, et al (2014) Progress towards genetics and breeding for minor genes based resistance to Ug99 and other rusts in CIMMYT high-yielding spring wheat. J Integr Agr 13:255–261.

[18] Rosewarne GM, Herrera-Foessel SA, Singh RP, Huerta-Espino J, Lan CX, He ZH (2013) Quantitative trait loci of stripe rust resistance in wheat. Theor Appl Genet 126:2427–2449. 10.1007/s00122-013-2159-9 23955314 PMC3782644

[19] Li ZF, Lan CX, He ZH, Singh RP, Rosewarne GM, Chen XM, et al (2014) Overview and application of QTL for adult plant resistance to leaf rust and powdery mildew in wheat. Crop Sci 54:1907–1925.

[20] Johnson R (1992) Past, present and future opportunities in breeding for disease resistance, with examples from wheat. Euphytica 63:3–22.

[21] McIntosh RA, Dubcovsky J, Rogers WJ, Morris C, Appels R, Xia XC (2014) Catalogue of gene symbols for wheat: 2013–2014 supplement, Available: http://www.shigen.nig.ac.jp/wheat/komugi/genes/macgene/supplement 2013. pdf.

[22] Ren Y, He ZH, Li J, Lillemo M, Wu L, Bai B, et al (2012) QTL mapping of adult-plant resistance to stripe rust in a population derived from common wheat cultivars Naxos and Shanghai3/Catbird. Theor Appl Genet 125:1211–1221. 10.1007/s00122-012-1907-6 22798057

[23] Devos KM, Millan T, Gale MD (1993) Comparative RFLP maps of the homoeologous group-2 chromosomes of wheat, rye and barley. Theor Appl Genet 85:784–792. 10.1007/BF00225020 24196051

[24] Suenaga K, Singh RP, Huerta-Espino J, William HM (2003) Microsatellite markers for genes Lr34/Yr18 and other quantitative trait loci for leaf rust and stripe rust resistance in bread wheat. Phytopathology 93:881–890. 10.1094/PHYTO.2003.93.7.881 18943170

[25] Lagudah ES, McFadden H, Singh RP, Huerta-Espino J, Bariana HS, Spielmeyer W (2006) Molecular genetic characterization of the Lr34/Yr18 slow rusting resistance gene region in wheat.Theor Appl Genet 114:21–30. 17008991 10.1007/s00122-006-0406-z

[26] Rosewarne GM, Sing RP, Huerta-Espino J, William HM, Bouchet S, Cloutier S, et al (2006) Leaf tip necrosis, molecular markers and β1-proteasome subunits associated with the slow rusting resistance genes Lr46/Yr29. Theor Appl Genet 112:500–508. 16331478 10.1007/s00122-005-0153-6

[27] William HM, Singh RP, Huerta-Espino J, Palacios G, Suenaga K (2006) Characterization of genetic loci conferring adult plant resistance to leaf rust and stripe rust in spring wheat. Genome 49:977–990. 17036073 10.1139/g06-052

[28] Lagudah ES (2011) Molecular genetics of race non-specific rust resistance in wheat. Euphytica 179:81–91.

[29] Uauy C, Brevis JC, Chen XM, Khan I, Jackson L, Chicaiza O, et al (2005) High-temperature adult-plant (HTAP) stripe rust resistance gene Yr36 from Triticum turgidum ssp. dicoccoides is closely linked to the grain protein content locus Gpc-B1. Theor Appl Genet 112:97–105. 16208504 10.1007/s00122-005-0109-x

[30] Fu DL, Uauy C, Distelfeld A, Blechl A, Epstein L, Chen XM, et al (2009) A kinase-start gene confers temperature-dependent resistance to wheat stripe rust. Science 323:1357–1360. 10.1126/science.1166289 19228999 PMC4737487

[31] Lin F, Chen XM (2007) Genetics and molecular mapping of genes for race specific and all-stage resistance and non-specific high temperature adult-plant resistance to stripe rust in spring wheat cultivar Alpowa. Theor Appl Genet 114:1277–1287. 17318493 10.1007/s00122-007-0518-0

[32] Herrera-Foessel SA, Lagudah ES, Huerta-Espino J, Hayden MJ, Bariana HS, Singh D, et al (2011) New slow-rusting leaf rust and stripe rust resistance genes Lr67 and Yr46 in wheat are pleiotropic or closely linked. Theor Appl Genet 122: 239–249. 10.1007/s00122-010-1439-x 20848270

[33] Lowe I, Jankuloski LC, Chao SM, Chen XM, See D, Dubcovsky J (2011) Mapping and validation of QTL which confer partial resistance to broadly virulent post-2000 North American races of stripe rust in hexaploid wheat. Theor Appl Genet 123:143–157. 10.1007/s00122-011-1573-0 21455722 PMC4761445

[34] McIntosh RA, Dubcovsky J, Rogers WJ, Morris C, Appels R, Xia XC (2011) Catalogue of gene symbols for wheat: 2011 supplement, Available: http://www.shigen.nig.ac.jp/wheat/komugi/genes/macgene/supplement2011.pdf.

[35] Ren RS, Wang MN, Chen XM, Zhang ZJ (2012) Characterization and molecular mapping of Yr52 for high-temperature adult-plant resistance to stripe rust in spring wheat germplasm PI 183527. Theor Appl Genet 125:847–857. 10.1007/s00122-012-1877-8 22562146

[36] Basnet BR, Singh RP, Ibrahim AMH, Herrera-Foessel SA, Huerta-Espino J, Lan CX, et al (2013) Characterization of Yr54 and other genes associated with adult plant resistance to yellow rust and leaf rust in common wheat Quaiu 3. Mol Breeding 33:385–399.

[37] Zhou XL, Wang MN, Chen XM, Lu Y, Kang ZS, Jing JX (2014) Identification of Yr59 conferring high-temperature adult-plant resistance to stripe rust in wheat germplasm PI178759. Theor Appl Genet 127:935–945. 10.1007/s00122-014-2269-z 24487945

[38] Lu Y, Wang MN, Chen XM, See D, Chao XM, Jing JX (2014) Mapping of Yr62 and a small-effect QTL for high-temperature adult-plant resistance to stripe rust in spring wheat PI 192252. Theor Appl Genet 127:1449–1459. 10.1007/s00122-014-2312-0 24781075

[39] Botstein DR, White RL, Skolnick M (1980) Construction of a genetic linkage map in man using restriction fragment length polymorphism. Am J Hum Genet 32:314–319. 6247908 PMC1686077

[40] Singh RP, Nelson JC, Sorrells ME (2000) Mapping Yr28 and other genes for resistance to stripe rust in wheat. Crop Sci 40:1148–1155.

[41] Velappan N, Snodgrass JL, Hakovirta JR, Marronea BL, Burde S (2001) Rapid identification of pathogenic bacteria by single-enzyme amplified fragment length polymorphism analysis. Diagn Micr Infec Dis 39:77–83. 10.1016/s0732-8893(00)00235-2 11248519

[42] Dedryver F, Paillard S, Mallard S, Robert O, Trottet M, Nègre S, et al (2009) Characterization of genetic components involved in durable resistance to stripe rust in the bread wheat Renan. Phytopathology 99:968–973. 10.1094/PHYTO-99-8-0968 19594316

[43] Röder MS, Korzun V, Wendehake K, Plaschke J, Tixier MH, Leroy P, et al (1998) A microsatellite map of wheat. Genetics 149:2007–2023. 9691054 10.1093/genetics/149.4.2007 PMC1460256

[44] Somers DJ, Isaac P, Edwards K (2004) A high-density micro-satellite consensus map for bread wheat (Triticum aestivum L.). Theor Appl Genet 109:1105–1114. 15490101 10.1007/s00122-004-1740-7

[45] Ren Y, Li ZF, He ZH, Wu L, Bai B, Lan CX, et al (2012) QTL mapping of adult-plant resistance to stripe rust and leaf rust in Chinese wheat cultivar Bainong 64. Theor Appl Genet 125:1253–1262. 10.1007/s00122-012-1910-y 22806327

[46] Wenzl P, Carling J, Kudrna D, Jaccoud D, Huttner E, Kleinhofs A, et al (2004) Diversity arrays technology (DArT) for whole-genome profiling of barley. Proc Natl Acad Sci USA 101:9915–9920. 15192146 10.1073/pnas.0401076101 PMC470773

[47] Avni R, Nave M, Eilam T, Sela H, Alekperov C, Peleg Z, et al (2014) Ultra-dense genetic map of durum wheat × wild emmer wheat developed using the 90K iSelect SNP genotyping assay. Mol Breeding 34:1549–1562.

[48] Colasuonno P, Gadaleta A, Giancaspro A, Nigro D, Giove S, Incerti O, et al (2014) Development of a high-density SNP-based linkage map and detection of yellow pigment content QTLs in durum wheat. Mol Breeding 34:1563–1578.

[49] Wang S, Wong D, Forrest K, Allen A, Chao S, Huang BE, et al (2014) Characterization of polyploid wheat genomic diversity using the high-density 90,000 SNP array. Plant Biotech J. 12:787–796. 10.1111/pbi.12183 PMC4265271 24646323

[50] Russo MA, Ficco DBM, Laido G, Marone D, Papa R, Blanco A, et al (2014) A dense durum wheat × T. dicoccum linkage map based on SNP markers for the study of seed morphology. Mol Breeding 34:1579–1597.

[51] Wang ZL, Li LH, He ZH, Duan XY, Zhou YL, Chen XM, et al (2005) Seedling and adult plant resistance to powdery mildew in Chinese bread wheat cultivars and lines. Plant Dis 89:457–463 10.1094/PD-89-0457 30795421

[52] Saghai-Maroof MA, Soliman KM, Jorgensen RA, Allard RW (1984) Ribosomal DNA spacer-length polymorphisms in barley: Mendelian inheritance, chromosomal location, and population dynamics. Proc Natl Acad Sci USA 81:8014–8018. 6096873 10.1073/pnas.81.24.8014 PMC392284

[53] Stam P (1993) Construction of integrated genetic linkage maps by means of a new computer package: JoinMap. Plant J 3:739–744.

[54] Voorrips RE (2002) MapChart: software for the graphical presentation of linkage maps and QTLs. J Hered 93:77–78. 12011185 10.1093/jhered/93.1.77

[55] Kosambi DD (1943) The estimation of map distance from recombination values. Annu Eugen 12:172–175.

[56] Wang S, Basten CJ, Zeng ZB (2006) Windows QTL Cartographer 2.5 Department of Statistics, North Carolina State University, Raleigh. Available: http://statgen.ncsu.edu/qtlcart/WQTLCart.htm.

[57] Rosewarne GM, Sing RP, Huerta-Espino J, Herrera-Foessel SA, Forrest KL, Hayden MJ, et al (2012) Analysis of leaf and stripe rust severities reveals pathotype changes and multiple minor QTLs associated with resistance in an Avocet × Pastor wheat population. Theor Appl Genet 124:1283–1294. 10.1007/s00122-012-1786-x 22274764

[58] Vazquez MD, Peterson CJ, Riera-Lizarazu O, Chen XM, Heesacker A, Ammar K, et al (2012) Genetic analysis of adult plant, quantitative resistance to stripe rust in wheat cultivar Stephens in multi-environment trials. Theor Appl Genet 124:1–11. 10.1007/s00122-011-1681-x 21912857

[59] Quan W, Hou GL, Chen J, Du ZY, Lin F, Guo Y, et al (2013) Mapping of QTL lengthening the latent period of Puccinia striiformis in winter wheat at the tillering growth stage. Eur J Plant Pathol 136:715–727.

[60] Khlestkina EK, Röder MS, Unger O, Meinel A, Börner A (2007) More precise map position and origin of a durable non-specific adult plant disease resistance against stripe rust (Puccinia striiformis) in wheat. Euphytica 153:1–10.

[61] Hao YF, Chen ZB, Wang YY, Bland D, Buck J, Brown-Guedira G, et al (2011) Characterization of a major QTL for adult plant resistance to stripe rust in US soft red winter wheat. Theor Appl Genet 123:1401–1411. 10.1007/s00122-011-1675-8 21830107

[62] Spielmeyer W, Sharp PJ, Lagudah ES (2003) Identification and validation of markers linked to broad-spectrum stem rust resistance gene Sr2 in wheat (Triticum aestivum L.). Crop Sci 43:333–336.

[63] Hare RA, McIntosh RA (1979) Genetic and cytogenetic studies of durable adult-plant resistances in Hope and related cultivars to wheat rusts. Zeitschrift für Pflanzenzüchtung 83:350–367.

[64] Cuthbert PA, Somers DJ, Thomas J, Cloutier S, Brulé-Babel A (2006) Fine mapping Fhb1, a major gene controlling Fusarium head blight resistance in bread wheat (Triticum aestivum L.). Theor Appl Genet 112:1465–1472. 16518614 10.1007/s00122-006-0249-7

[65] Mago R, Tabe L, McIntosh RA, Pretorius Z, Kota R, Paux E (2011) A multiple resistance locus on chromosome arm 3BS in wheat confers resistance to stem rust (Sr2), leaf rust (Lr27) and powdery mildew. Theor Appl Genet 123:615–623. 10.1007/s00122-011-1611-y 21573954

[66] Imtiaz K, Ahmad M, Cromey MG, Griffin WB, Hampton JG (2004) Detection of molecular markers linked to the durable adult plant stripe rust resistance gene Yr18 in bread wheat (Triticum aestivum L.). Plant Breeding 123: 401–404.

[67] Zwart RS, Thompson JP, Milgate AW, Bansal UK, Williamson PM, Raman H. et al (2010) QTL mapping of multiple foliar disease and root-lesion nematode resistances in wheat. Mol Breeding 26:107–124.

[68] Dyck PL (1987) The association of a gene for leaf rust resistance with the chromosome 7D suppressor of stem rust resistance in common wheat. Genome 29: 467–469.

[69] McIntosh RA (1992) Close genetic linkage of genes conferring adult-plant resistance to leaf rust and stripe rust in wheat. Plant Pathol 41:523–527.

[70] Singh RP (1992) Genetic association of leaf rust resistance gene Lr34 with adult plant resistance to stripe rust in bread wheat. Phytopathology 82:835–838.

[71] Bariana HS, Hayden MJ, Ahmed NU, Bell JA, Sharp PJ, McIntosh RA (2001) Mapping of durable adult plant and seedling resistances to stripe rust and stem rust diseases in wheat. Aust J Agr Res 52:1247–1255.

[72] Bariana HS (2010) QTL mapping of multiple foliar disease and root-lesion nematode resistances in wheat. Mol Breeding 26:107–124.

[73] Boukhatem N, Baret PV, Mingeot D, Jacquemin JM (2002) Quantitative trait loci for resistance against yellow rust in two wheat derived recombinant inbred line populations. Theor Appl Genet 104:111–118. 12579435 10.1007/s001220200013

[74] Ramburan VP, Pretorius ZA, Louw JH, Boyd LA, Smith PH, Boshoff WHP, et al (2004) A genetic analysis of adult plant resistance to stripe rust in the wheat cultivar Kariega. Theor App Genet 108:1426–1433. 10.1007/s00122-003-1567-7 14963651

[75] Navabi A, Tewari JP, Singh RP, McCallum B, Laroche A, Briggs KG (2005) Inheritance and QTL analysis of durable resistance to stripe and leaf rusts in an Australian cultivar, Triticum aestivum Cook. Genome 48:97–107. 15729401 10.1139/g04-100

[76] Lillemo M, Asalf B, Singh RP, Huerta-Espino J, Chen XM, He ZH, et al (2008) The adult plant rust resistance loci Lr34/Yr18 and Lr46/Yr29 are important determinants of partial resistance to powdery mildew in bread wheat line Saar. Theor Appl Genet 116:1155–1166. 10.1007/s00122-008-0743-1 18347772

[77] Akhunov E, Nicolet C, Dvorak J (2009) Single nucleotide polymorphism genotyping in polyploid wheat with the Illumina GoldenGate assay. Theor Appl Genet 119:507–517. 10.1007/s00122-009-1059-5 19449174 PMC2715469

[78] Ren J, Sun D, Chen L, You FM, Wang J, Peng Y, et al (2013) Genetic diversity revealed by single nucleotide polymorphism markers in a worldwide germplasm collection of durum wheat. Int J Mol Sci 14:7061–7088. 10.3390/ijms14047061 23538839 PMC3645677

[79] Pei D, Zhang W, Sun H, Wei X, Yue J, Wang H (2014). Identification of autophagy-related genes ATG4 and ATG8 from wheat (Triticum aestivum L.) and profiling of their expression patterns responding to biotic and abiotic stresses. Plant Cell Rep, 33:1697–1710 10.1007/s00299-014-1648-x 24996626

[80] Jia J, Zhao S, Kong X, Li Y, Zhao G, He W, et al (2013). Aegilops tauschii draft genome sequence reveals a gene repertoire for wheat adaptation. Nature, 496:91–95. 10.1038/nature12028 23535592

[81] Lan CX, Zhang YL, Herrera-Foessel SA, Basnet BR, Huerta-Espino J, Lagudah ES, et al (2015) Identification and characterization of pleiotropic and co-located resistance loci to leaf rust and stripe rust in bread wheat cultivar Sujata. Theor Appl Genet 128:549–561. 10.1007/s00122-015-2454-8 25613742

[82] Veldboom LR, Lee M (1996) Genetic mapping of quantitative trait loci in maize in stress and non-stress environments: I. Grain yield and yield components. Crop Sci 36:1310–1319.

[83] Semagn K, Babu R, Hearne S, Olsen M (2014) Single nucleotide polymorphism genotyping using Kompetitive Allele Specific PCR (KASP): overview of the technology and its application in crop improvement. Mol breeding 33:1–14.

