# Supplementary figures and images for: Genome-Wide Linkage Mapping of QTL for Adult-Plant Resistance to Stripe Rust in a Chinese Wheat Population Linmai 2 × Zhong 892
Source: PLoS One. 2015 Dec 29;10(12):e0145462. doi: 10.1371/journal.pone.0145462 (PMC4694644; doi:10.1371/journal.pone.0145462)

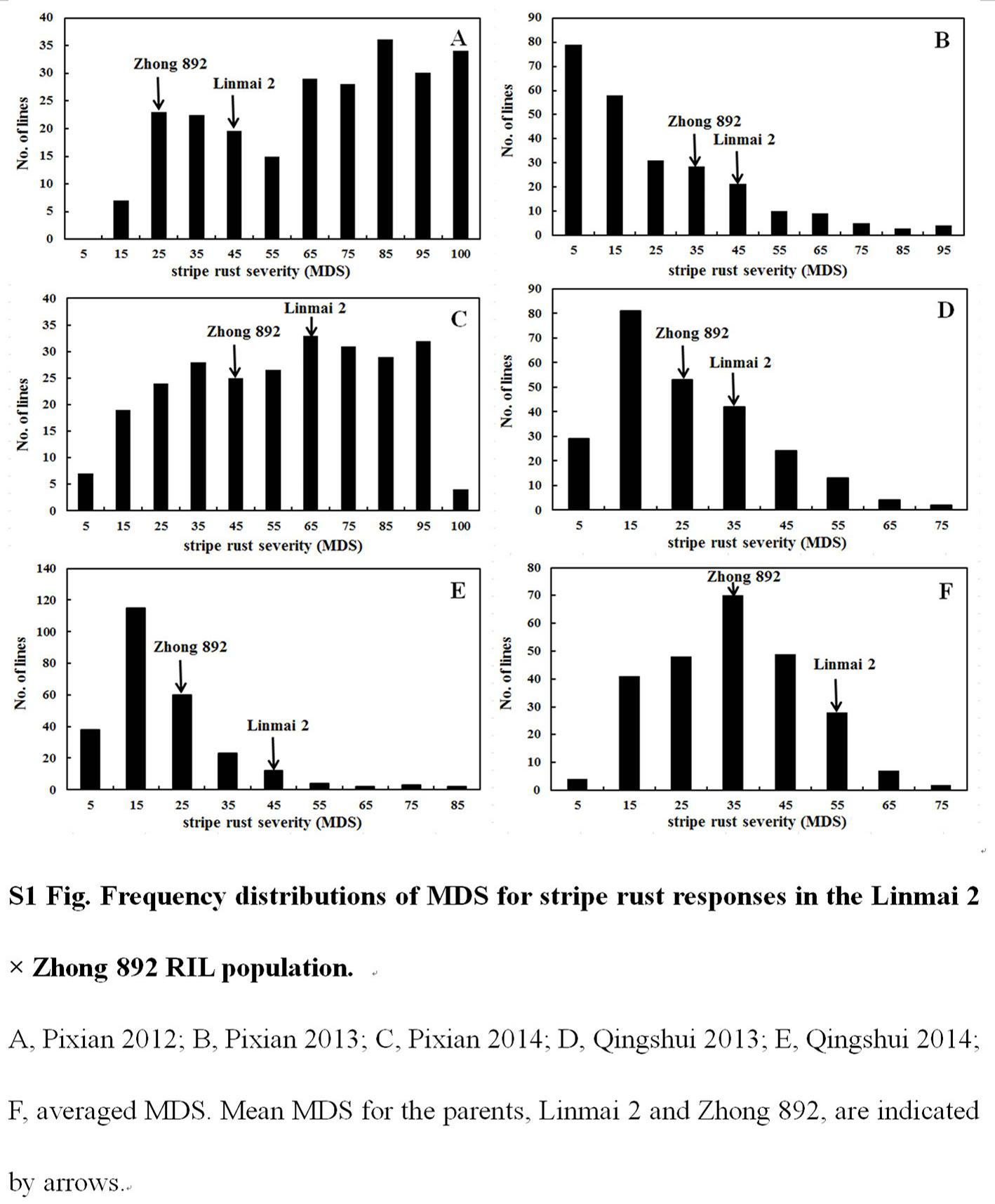

Supplement: S1 Fig — A, Pixian 2012; B, Pixian 2013; C, Pixian 2014; D, Qingshui 2013; E, Qingshui 2014; F, averaged MDS. Mean MDS for the parents, Linmai 2 and Zhong 892, are indicated by arrows. (TIF) [file pone.0145462.s001.tif]
