# Supplementary material for: Genome-Wide Linkage Mapping of QTL for Adult-Plant Resistance to Stripe Rust in a Chinese Wheat Population Linmai 2 × Zhong 892
Source: PLoS One. 2015 Dec 29;10(12):e0145462. doi: 10.1371/journal.pone.0145462 (PMC4694644; doi:10.1371/journal.pone.0145462)
Supplement: S1 Table — (DOCX) [file pone.0145462.s002.docx]

**S1 Table. Mean stripe rust severity of lines with different QTL combinations from the Linmai 2/Zhong 892 RIL population.**

| **Genotypic classes** | **RILs (No.)** | **MDS (%)** | | | | | |
| --- | --- | --- | --- | --- | --- | --- | --- |
|  |  | **Pixian 2012** | **Pixian 2013** | **Pixian 2014** | **Qingshui 2013** | **Qingshui 2014** | **Average** |
| none | 9 | 79.2 A | 82.6 A | 88.3 A | 72.6 A | 73.9 A | 79.3 A |
| 2AL | 4 | 78.3 A | 70.3 A | 86.1 A | 64.1 A | 60.9 A | 72.8 A |
| 7DS | 4 | 73.6 A | 62.9 A | 84.8 A | 54.3 B | 61.3 A | 69.4 A |
| 2AL+7DS | 2 | 70.2 A | 73.6 A | 80.5 A | 56.6 B | 55.6 B | 68.8 A |
| 7AL | 5 | 72.6 A | 62.3 A | 80.3 A | 61.2 A | 58.9 B | 68.4 A |
| 5DL | 4 | 70.5 A | 63.5 A | 85.2 A | 51.2 B | 60.3 A | 67.1 A |
| 5DL+6AL | 3 | 60.3 A | 68.9 A | 78.5 A | 45.9 B | 48.9 B | 63.0 A |
| 2AL+2BL | 2 | 68.9 A | 58.8 B | 76.9 A | 50.3 B | 42.9 B | 61.0 A |
| 3AS | 5 | 63.2 A | 58.6 B | 70.2 A | 48.9 B | 58.3 B | 60.6 A |
| 2BL+3AS | 3 | 58.2 B | 66.3 A | 72.4 A | 55.2 B | 50.3 B | 60.5 A |
| 3BS+7AL+7DS | 5 | 56.3 B | 69.3 A | 77.5 A | 48.9 B | 38.5 C | 58.0 A |
| 3AS+5DL+7AL | 3 | 60.2 A | 65.3 A | 70.3 A | 35.2 C | 53.6 B | 56.9 A |
| 3BS+5DL | 5 | 58.9 B | 50.3 B | 72.5 A | 39.6 C | 50.8 B | 56.8 A |
| 2AL+2BL+5DL | 5 | 65.3 A | 55.5 B | 63.2 A | 43.2 B | 52.0 B | 56.2 A |
| 3AS+5DL+6AL | 4 | 54.2 B | 50.2 B | 69.3 A | 48.9 B | 55.3 B | 55.5 A |
| 3AS+7DS | 5 | 55.6 B | 60.3 A | 68.9 A | 40.2 B | 52.3 B | 55.5 A |
| 5DL+6AL+7DS | 3 | 63.9 A | 55.2 B | 55.3 B | 50.3 B | 48.9 B | 55.3 A |
| 3BS+6AL | 2 | 60.3 A | 38.9 C | 65.6 A | 46.8 B | 53.3 B | 52.9 A |
| 5DL+7AL+7DS | 5 | 68.9 A | 49.2 B | 70.6 A | 40.3 B | 45.8 B | 54.8 A |
| 2AL+5DL+7AL | 5 | 55.6 B | 61.2 A | 70.3 A | 40.3 B | 38.9 C | 54.6 A |
| 3AS+5DL | 4 | 57.5 B | 50.6 B | 68.8 A | 39.2 C | 50.5 B | 54.5 A |
| 2AL+5DL+7DS | 3 | 52.9 B | 60.3 A | 68.9 A | 40.6 B | 48.9 B | 54.3 A |
| 5DL+7AL | 4 | 62.5 A | 48.3 B | 58.3 B | 52.6 B | 46.3 B | 53.1 A |
| 5DL+7DS | 4 | 39.8 C | 50.3 B | 62.5 A | 48.0 B | 52.9 B | 52.3 A |
| 6AL+7DS | 4 | 52.3 B | 58.9 B | 65.3 A | 39.5 C | 48.9 B | 53.0 A |
| 3BS+6AL+7DS | 4 | 60.3 A | 39.5 C | 69.5 A | 45.2 B | 43.9 B | 51.7 A |
| 3BS+7DS | 2 | 48.9 B | 53.6 B | 62.8 A | 41.5 B | 41.3 B | 51.4 A |
| 5DL+6AL+7AL+7DS | 3 | 60.2 A | 45.6 B | 69.3 A | 35.9 C | 42.3 B | 50.7 A |
| 3BS+5DL+7AL | 4 | 51.3 B | 44.6 B | 65.8 A | 40.3 B | 50.3 B | 50.5 A |
| 2AL+2BL+6AL+7AL | 3 | 53.2 B | 51.6 B | 60.3 A | 35.3 C | 44.9 B | 49.0 B |
| 2AL+3AS+5DL+7DS | 5 | 50.3 B | 45.2 B | 61.5 A | 38.9 C | 43.9 B | 49.0 B |
| 2AL+3AS+3BS+5DL | 5 | 52.9 B | 44.6 B | 60.3 A | 38.9 C | 45.4 B | 48.4 B |
| 2AL+3AS | 4 | 54.3 B | 49.8 B | 36.5 C | 44.3 B | 48.3 B | 48.2 B |
| 2AL+3AS+6AL | 2 | 50.2 B | 45.6 B | 58.3 B | 35.5 C | 48.3 B | 47.6 B |
| 3AS+7AL+7DS | 3 | 48.9 B | 55.6 B | 50.3 B | 32.8 C | 48.9 B | 47.3 B |
| 3AS+5DL+6AL+7AL | 3 | 45.5 B | 52.3 B | 61.2 A | 32.5 C | 42.9 B | 46.9 B |
| 2AL+3AS+7DS | 3 | 35.8 C | 45.3 B | 55.8 B | 46.6 B | 50.9 B | 46.9 B |
| 2AL+3BS+5DL+7AL | 2 | 51.2 B | 42.3 B | 55.9 B | 35.8 C | 48.9 B | 46.8 B |
| 6AL+7Al+7DS | 4 | 48.9 B | 38.9 C | 60.3 A | 35.5 C | 50.2 B | 46.8 B |
| 3AS+3BS+7DS | 3 | 38.2 C | 38.6 C | 60.3 A | 43.2 B | 53.3 B | 46.7 B |
| 2AL+6AL+7AL+7DS | 3 | 56.2 B | 40.3 B | 53.6 B | 36.8 C | 40.5 B | 45.4 B |
| 2AL+3BS+5DL+6AL | 1 | 56.7 B | 40.3 B | 48.6 B | 33.9 C | 42.5 B | 44.4 B |
| 3AS+3BS | 3 | 39.5 C | 51.2 B | 55.6 B | 33.6 C | 40.2 B | 44.0 B |
| 3AS+6AL+7AL+7DS | 4 | 40.3 B | 45.9 B | 55.9 B | 34.9 C | 42.8 B | 44.0 B |
| 2AL+3AS+5DL+6AL+7DS | 6 | 50.2 B | 44.6 B | 51.2 B | 35.6 C | 43.6 B | 43.7 B |
| 3AS+3BS+7AL | 2 | 45.6 B | 32.9 C | 56.9 B | 33.6 C | 35.6 C | 43.4 B |
| 3AS+3BS+6AL | 2 | 41.2 B | 50.3 B | 60.9 A | 30.2 C | 33.2 C | 43.2 B |
| 2AL+3AS+3BS | 2 | 39.5 C | 44.9 B | 51.2 B | 32.5 C | 45.6 B | 42.7 B |
| 3BS+6AL+7AL+7DS | 2 | 56.9 B | 25.2 C | 61.3 A | 28.9 C | 40.3 B | 42.5 B |
| 2BL+3AS+3BS+7DS | 5 | 45.8 B | 33.6 C | 52.3 B | 38.9 C | 40.6 B | 42.2 B |
| 3AS+6AL+7DS | 4 | 31.9 C | 48.6 B | 55.3 B | 40.6 B | 32.8 C | 41.8 B |
| 3AS+3BS+6AL+7AL | 4 | 45.3 B | 33.6 C | 55.2 B | 36.5 C | 32.9 C | 40.7 B |
| 2BL+3BS+6AL+7AL+7DS | 2 | 40.6 B | 48.6 B | 44.3 B | 26.9 C | 39.6 C | 40.0 C |
| 3BS+5DL+7AL+7DS | 4 | 36.9 C | 40.6 B | 55.2 B | 26.9 C | 38.3 C | 39.6 C |
| 2AL+3BS+7AL+7DS | 4 | 38.5 C | 40.3 B | 48.9 B | 28.6 C | 41.2 B | 39.5 C |
| 3BS+5DL+6AL+7DS | 5 | 50.8 B | 32.6 C | 50.9 B | 22.5 C | 39.8 C | 39.3 C |
| 2AL+2BL+3BS+6AL+7AL | 4 | 25.4 C | 40.9 B | 48.9 B | 32.5 C | 45.0 B | 38.5 C |
| 2AL+3BS+5DL+6AL+7DS | 3 | 40.6 B | 32.3 C | 55.2 B | 26.9 C | 35.8 C | 38.2 C |
| 3AS+5DL+6AL+7DS | 1 | 32.5 C | 40.3 B | 51.2 B | 25.8 C | 36.9 C | 37.3 C |
| 3AS+5DL+6AL+7AL+7DS | 1 | 40.3 B | 36.5 C | 44.9 B | 23.2 C | 25.9 C | 36.9 C |
| 3AS+3BS+5DL+6AL | 1 | 35.6 C | 30.2 C | 53.2 B | 25.3 C | 40.2 B | 36.9 C |
| 3BS+5DL+6AL+7AL+7DS | 2 | 32.8 C | 40.3 B | 36.9 C | 25.8 C | 39.6 C | 36.1 C |
| 2AL+2BL+3AS+5DL+7AL | 4 | 50.3 B | 35.8 C | 40.6 B | 21.2 C | 31.9 C | 36.0 C |
| 2AL+3AS+3BS+6AL+7DS | 5 | 36.0 C | 33.2 C | 40.7 B | 35.3 C | 32.1 C | 35.5 C |
| 3AS+3BS+5DL+6AL+7DS | 3 | 28.9 C | 36.9 C | 45.3 B | 25.6 C | 39.6 C | 35.3 C |
| 2AL+3BS+5DL+7DS | 5 | 30.6 C | 25.8 C | 45.3 B | 32.6 C | 40.2 B | 34.9 C |
| 3AS+3BS+5DL+7DS | 6 | 25.2 C | 30.6 C | 48.3 B | 28.3 C | 36.9 C | 33.9 C |
| 3AS+3BS+5DL+7AL+7DS | 2 | 20.5 C | 30.6 C | 45.3 B | 18.3 D | 36.9 C | 30.3 C |
| 2BL+3AS+3BS+5DL+6AL+7DS | 4 | 32.3 C | 25.6 C | 45.3 B | 18.5 D | 29.3 C | 30.2 C |
| 3AS+3BS+6AL+7AL+7DS | 3 | 17.5 D | 28.9 C | 36.3 C | 25.6 C | 38.6 C | 29.4 C |
| 2AL+2BL+3BS+6AL+7AL+7DS | 2 | 41.2 B | 32.1 C | 39.6 C | 12.8 D | 20.6 C | 29.3 C |
| 2AL+3AS+3BS+5DL+7AL+7DS | 2 | 23.9 C | 30.6 C | 33.6 C | 18.9 D | 36.9 C | 28.8 C |
| 2AL+3BS+5DL+6AL+7AL+7DS | 3 | 32.8 C | 28.6 C | 40.6 B | 9.8 E | 29.6 C | 28.3 C |
| 2AL+3AS+3BS+5DL+6AL+7AL+7DS | 3 | 25.6 C | 22.3 C | 37.9 C | 12.5 D | 22.4 C | 24.1 C |
| 2AL+2BL+3AS+3BS+5DL+7AL | 2 | 11.2 D | 19.8 D | 29.5 C | 19.3 D | 35.6 C | 23.3 C |
| 3AS+3BS+5DL+6AL+7AL+7DS | 2 | 13.9 D | 20.1 C | 35.6 C | 10.0 D | 25.3 C | 21.0 C |
| 2AL+2BL+3AS+3BS+5DL+6AL+7DS | 3 | 20.1 C | 15.8 D | 23.6 C | 5.9 E | 18.9 D | 16.8 D |
| 2AL+2BL+3AS+3BS+5DL+7AL+7DS | 2 | 15.4 D | 9.9 E | 12.3 D | 6.9 E | 31.8 D | 15.3 D |
| 2BL+3AS+3BS+5DL+6AL+7AL+7DS | 5 | 9.5 E | 18.3 D | 25.6 C | 4.9 E | 8.8 E | 13.4 D |

Different letters following the means indicate significant differences at *P* < 0.01
